# Enhanced Assembling of N-and-K-Riched Macroalgae as Carbon Adsorbent for CO_2_ Capture with Ni(NO_3_)_2_/KOH as Co-Catalysts

**DOI:** 10.3390/molecules28176242

**Published:** 2023-08-25

**Authors:** Huijuan Ying, Ganning Zeng, Yaohong He, Yanjun Hou, Ning Ai

**Affiliations:** 1 College of Chemical Engineering, Zhejiang University of Technology, Hangzhou 310014, China; yinghuijuan@zjut.edu.cn (H.Y.); 2112101359@zjut.edu.cn (Y.H.); hyj80565679@163.com (Y.H.); 2 College of Environment, Zhejiang University of Technology, Hangzhou 310014, China; gnzeng@zjut.edu.cn; 3 College of Biological, Chemical Science and Engineering, Jiaxing University, Jiaxing 314001, China

**Keywords:** microporous, KOH, macroalgae, CO_2_ adsorption

## Abstract

Porous-activated carbons have drawn great attention due to their important role in CO_2_ capture. Ni(NO_3_)_2_/KOH, as co-catalysts under different temperatures, were studied to obtain porous graphitized carbon from *Sargassum horneri* feedstock. The results indicated that the properties of the porous graphitized carbon generated at 850 °C were greatly enhanced, showing a large specific surface area of 1486.38 cm^3^·g^−1^ with narrowly distributed micropores (~0.67 nm) and abundant functional groups, which endowed high CO_2_ uptake; moreover, the high CO_2_ uptake was mainly attributed to the synergistic effect of Ni(NO_3_)_2_ and KOH, both in chemical modification and pore formation. The fitted values of the four kinetic models showed that the double exponential model provided the best description of carbon adsorption, indicating both physical and chemical adsorption. It is worth noting that carbon could be reused four times in the adsorption/desorption procedure in this research with good stability. This work focuses on the high-value-added comprehensive utilization of macroalgae, which not only is important for high-performance adsorbent preparation but also has positive benefits for the development and utilization of macroalgae resources.

## 1. Introduction

To alleviate food shortage and meet the world’s energy demands, increasing attention is being paid to the utilization of various marine biomasses. Macroalgae, a kind of marine biomass that can be easily cultivated with no need for land or fertilizer, has quick growth cycles, is of low cost, and is considered a promising and long-term precursor to ensure the availability of raw materials for large-scale industrial utilizations. Various products are prepared from macroalgae and applied in fields such as biomedicine and electrochemistry, the bio-fuel industry, and sewage treatment, and all of these productions have shown good performance [1,2,3,4,5]; however, to date, it is estimated that <1% of the total marine biomass has been utilized for human activities, presenting enormous untapped opportunities. Meanwhile, there are also some negative stories about macroalgae. Since 2011, the continuous explosion of golden tides caused by *Sargassum horneri* (SH) in West Africa and in the Yellow Sea of China has ceaselessly destroyed coastal ecosystems, leading to serious economic loss due to its terrible influence on water quality, ocean transportation, and tourism [6,7]. The Caribbean-wide SH clean-up in 2018 cost USD 120 million, exclusive of the decreased revenues from the lost tourism. SH biomass strandings can also cause respiratory issues during their decaying process and other human health problems, such as increased vibrio bacteria [8]; therefore, it is necessary to take advantage of this emerging abundance of biomass strandings of SH effectively.

SH is artificially cultivated for aquatic eutrophication restoration due to its superior absorption capability of metal cations (i.e., Ni^2+^, Fe^3+^, and Mg^2+^) to form metal alginates (M-alginates) via the alginate polymers on its cell walls. These metal cations have been proven to be capable of synthesizing special carbon materials with a porous structure, and they can be easily removed via acid washing after carbonization [9,10,11]. Meanwhile, the disordered structure of biochar can be converted to graphitized carbon materials using metal catalysts at moderate temperatures to form specific functional groups [12,13]. Therefore, it is possible to reduce the amount of additional metals needed, or even eliminate the need for additional metal entirely, if SH is used as a precursor. On the other hand, SH is also a kind of protein-rich marine biomass, which means it has abundant nitrogen that can lead to a self-modulated nitrogen-doping effect. Recently, a new report pointed out that the nitrogen contents in SH continue to increase [14]. Thus, by choosing SH as a precursor, the serious economic loss caused by the occurrence of massive golden tides can be avoided, and this can also give full play to its own advantages in preparing high-value-added products.

Generally, carbon materials prepared via direct carbonization cannot meet the actual demand. An activation step is always required for the generation of activated carbon as the chemical process can benefit from the generation of significant porous structures and considerable function groups on the surface of carbon [15]. Among the common chemical agents, such as ZnCl_2_, FeCl_3_, KOH, K_2_CO_3_, and H_3_PO_4_, KOH has been widely used to prepare activated carbon with good performance [16,17,18,19]; however, the need for a massive dose of KOH is still a hindrance to up-scale utilization. Considering that SH is a high potassium species, it is possible to reduce the amount of KOH to prepare activated carbon. On the other hand, inspired by the synergistic effect of metal salts and KOH in carbon activation [12,20], Ni(NO_3_)_2_·6H_2_O was added to obtain a higher yield with more graphitized carbon materials, to reduce the dosage of KOH needed under lower activation temperatures to form well-developed porous structures. Meanwhile, it was proven that Ni(NO_3_)_2_ was easier to remove in previous research because it could be decomposed into Ni oxides, which were subsequently reduced to metallic Ni at elevated temperatures [21,22], while the transition metals Fe and Co could form FeN_x_ and CoN_x_ composites [23,24] with the introduction of a N-containing substance.

In this study, SH was selected as the precursor, and a porous graphitized carbon was synthesized with a fixed Ni(NO_3_)_2_/KOH co-catalyst dosage under different activation temperatures. The activated carbon that was generated was characterized and applied to the process of CO_2_ capture. The adsorption performance of CO_2_ was carefully investigated at incremental temperatures. The kinetic mechanism underlying the adsorption of CO_2_ in the generated porous graphitized carbon was also studied, and the cyclic adsorption/desorption behavior was assessed to evaluate the composite’s feasibility for long-term application. The activated carbon generated via Ni(NO_3_)_2_/KOH activation from N-and-K-riched macroalgae opens a new horizon and has great potential application in CO_2_ capture in the future.

## 2. Results and Discussion

### 2.1. Material Characterization

#### 2.1.1. Component Analysis of the Samples

The elemental analysis results of SH with different biomasses are listed in Table 1. As shown in Table 1, the C content of SH is lower than those of the terrestrial biomasses but slightly higher than those of other marine biomasses, while the H contents of all biomasses are similar. Our original intention was to turn carbon precursors from terrestrial biomasses to marine biomasses and resolve the golden tide effects; therefore, choosing SH from these three kinds of macroalgae as the carbon precursor is a good decision to obtain a higher carbon yield. In addition, the N content of SH is the highest among all biomasses, which may provide high-quality N-doped activated carbons with special surface functional groups that can enhance the CO_2_ uptake capacity. This was further verified via FTIR and XPS spectroscopy. It is interesting that the K content of SH is the highest, reaching 100 times more than that of the terrestrial biomass Populus wood. Studies have shown that K species can etch carbon frameworks to generate a pore network at high temperatures. The formed K plays a vital role in forming a large specific surface area because it can intercalate into the carbon lattice to make it expand, which is unable to recover even after the formed K has been washed away [16]. Generally, this process can be described using Equations (1)–(5). Therefore, SH has the possibility of self-assembly in this sense. This was confirmed through subsequent comparative experiments.

6KOH + 2C → 2K + 3H_2_ + 2K_2_CO_3_ (1)

K_2_CO_3_ → K_2_O + CO_2_ (2)

CO_2_ + C → 2CO (3)

K_2_CO_3_ + 2C → 2K + 3CO (4)

K_2_O + C → 2K + CO (5)

The elemental compositions of SH, SH-850, and ASH-850 are presented in Table 2. After Ni(NO_3_)_2_/KOH activation, the relative carbon content of the samples significantly increased from 29.25% to 84.24%, while the nitrogen content exhibited a decrease, which might be related to the decomposition of unstable nitrogen-containing groups under high temperatures; however, they could form useful nitrogenous functional groups on the surface of ASH-850, which could generate more active sites to improve the CO_2_ adsorption capacity. In addition, the hydrogen content also decreased, probably because hydrogen turns into water steam at high temperatures via the oxidizing reaction. These series of redox reactions can generate a pore network and enhance the porosity of the samples, which is also helpful for CO_2_ capture.

#### 2.1.2. Morphological Analysis of the Samples

**Figure 1:**
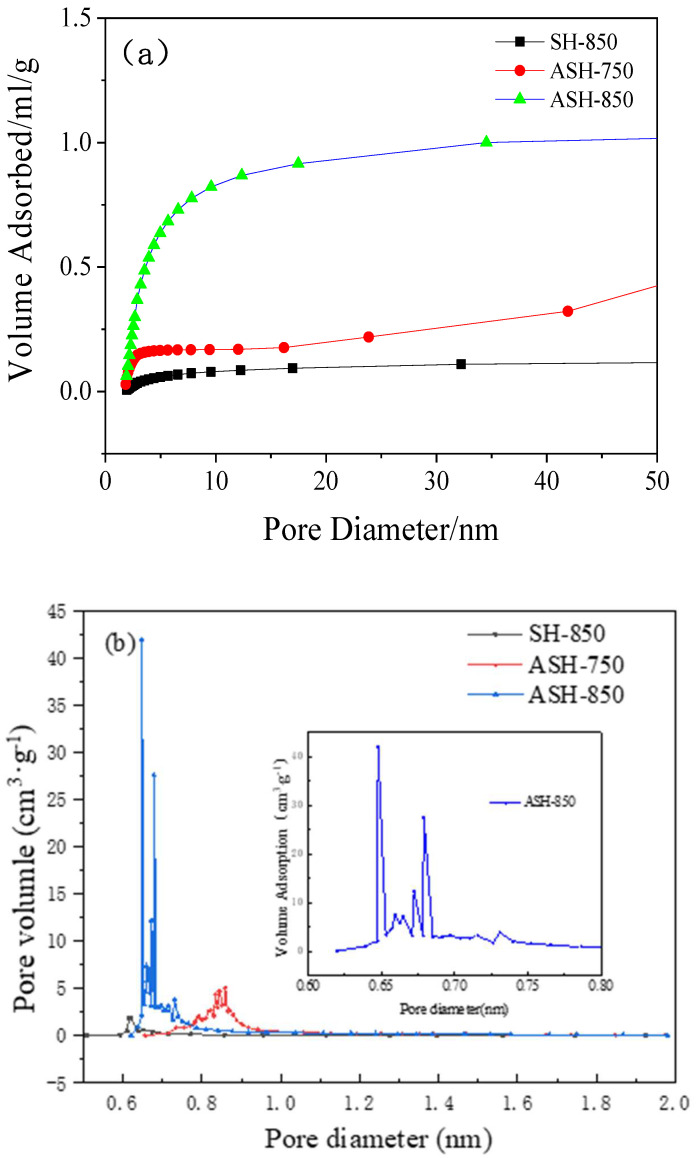
N_2_ adsorption–desorption isotherm results of the samples: (**a**) whole-pore-size distribution, and (**b**) micropore-size distribution.

**Figure 2:**
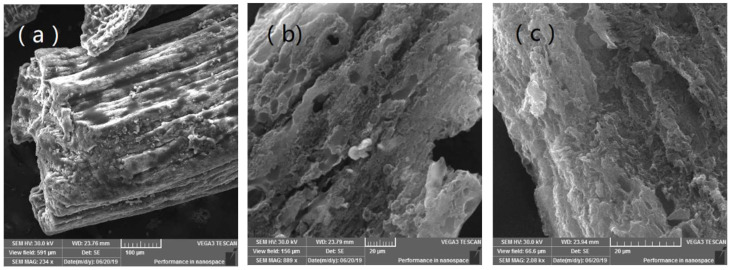

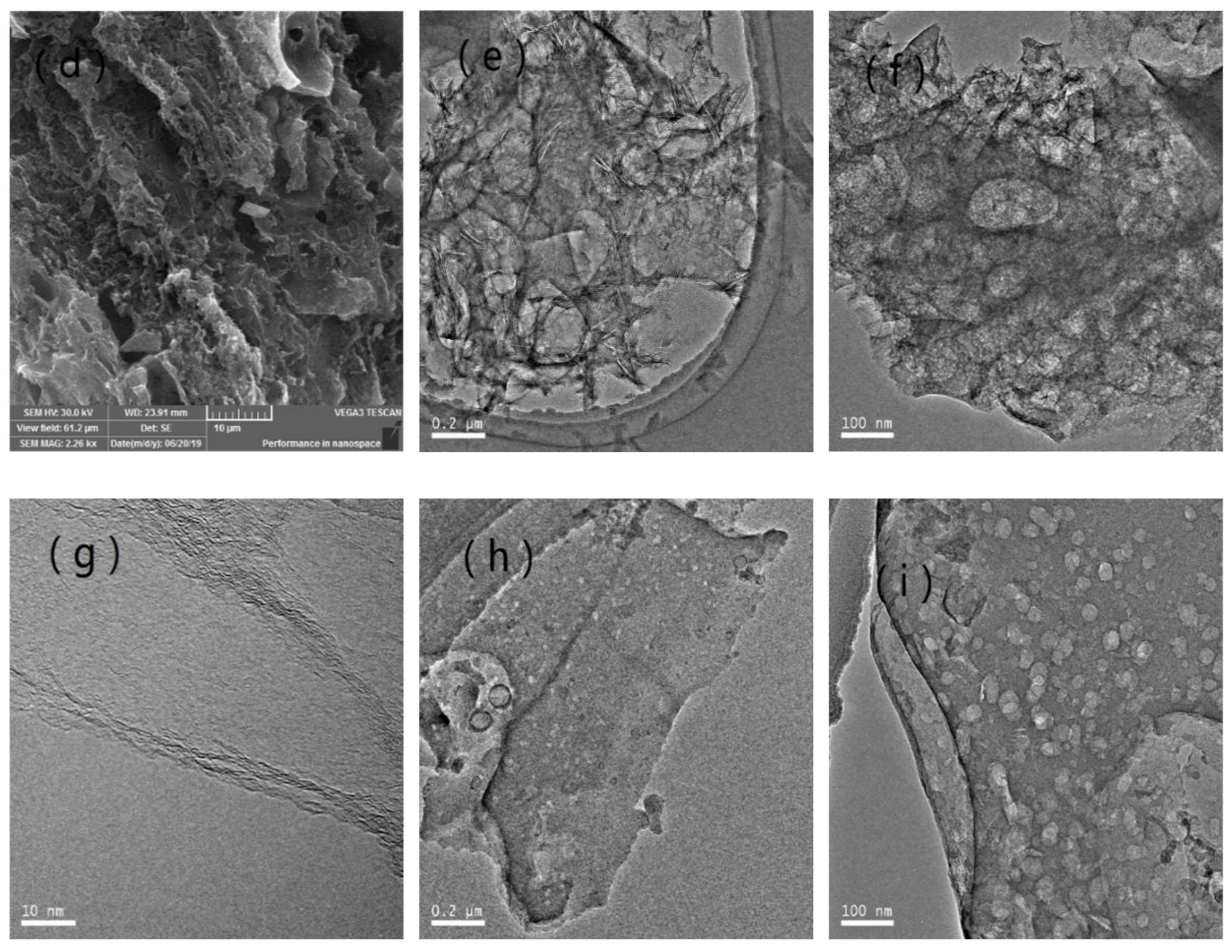
SEM and TEM of SH, ASH-750, and ASH-850: (**a**) SEM of SH; (**b**) SEM of ASH-750; (**c**,**d**) SEM of ASH-850; (**e**,**f**) TEM with different magnifications of ASH-750; and (**g**–**i**) TEM with different magnifications of ASH-850.

Figure 1 and Table 3 show the N_2_ sorption–desorption isotherms of the samples and their pore-size distribution. It can be seen from Table 3 that SH-850 and ASH-650 both have a low surface area, while others all have large surface areas of more than 1000 m^2^·g^−1^. Meanwhile, SH-850 still has a surface area of 133 m^2^·g^−1^, with an average pore size of 4.3 nm. This demonstrates that SH can self-assemble under the activation process, which indicates that the dosage of KOH can be reduced. This is consistent with our assumptions. It is worth mentioning that the surface area of ASH-850 is the highest and that of ASH-750 is the second highest, at 1486 m^2^·g^−1^ and 1307 m^2^·g^−1^, respectively. It is interesting that the largest total pore volume of the activated carbon we obtained is 2.27 cm^3^·g^−1^ in ASH-750, which is twice that of ASH-850; however, about only one-third micropore volume was found in ASH-750, while nearly 80% micropore volume was found in ASH-850. This is shown in Figure 1a, where we can find all three types (micro-, meso-, and macro-pores) of pores in ASH-750, while ASH-850 presents a relatively narrow microporous structure (~0.67 nm), as shown in Figure 1b. These representative porous structures were further confirmed using scanning electron microscopy (SEM). The original sample SH has a tubular pipe shape without any porous structure (Figure 2a). On the other hand, Figure 2b–d display a completely different morphology, with a high number of holes developed due to preferential etching by activator KOH in the amorphous portions of carbon. In order to see the nanostructures more clearly, characterization using transmission electron microscopy (TEM) was performed, and the results are shown in Figure 2e–i. We can see wrinkled nanostructures, nano-porous structures, and high graphitic carbon structures. Specifically, there are wrinkled layers in both samples of ASH-750 and ASH-850 but a diversity of connected porous structures is found in ASH-750 (Figure 2e,f), and massive micropores with few macro-porous structures can be seen in ASH-850 (Figure 2h,i). These results are in good agreement with the data of BET and SEM. In addition, the ASH-850 sample is composed of filmy carbon layers with about 10 layers being homogeneously stacked together, and a microporous structure with a narrow size is found in every layer (Figure 2h). These micropores play a key role in increasing the adsorption of CO_2_. The main factor is that the rich, narrow micropores can quickly enhance the interaction energy of CO_2_ molecules and adsorbent due to the overlapping of the potential fields from adjacent walls [28].

#### 2.1.3. Characterization of the Samples Using Fourier-Transform Infrared Spectra

**Figure 3:**
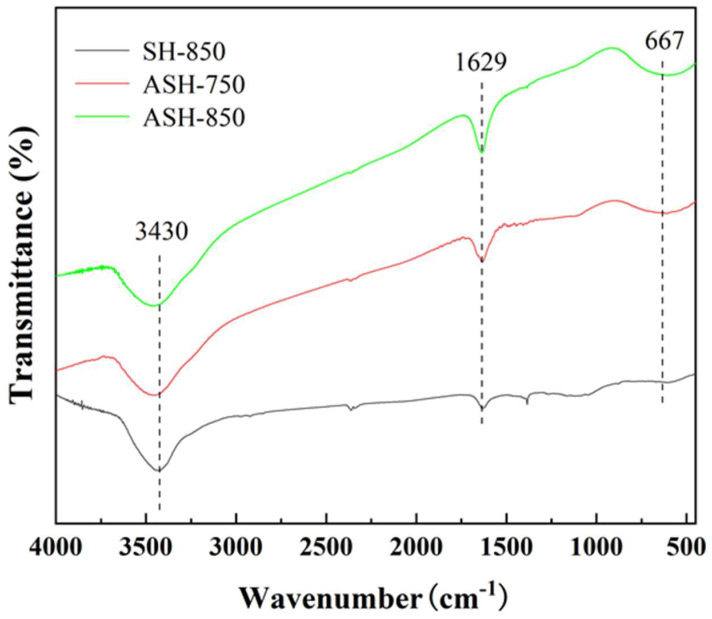
FTIR spectra of the SH-850, ASH-750, and ASH-850 samples.

The infrared spectral results are shown in Figure 3. The broad absorption peak between 500 cm^−1^ and 800 cm^−1^ corresponds to the N-H deformation vibration. These nitrogenous functional groups might originate from the N-riched precursor SH, and they are beneficial for CO_2_ capture. Meanwhile, there are absorption peaks near 3430 cm^−1^ (corresponding to the O-H stretching vibration of hydroxyl and carboxyl groups) and absorption peaks at about 1629 cm^−1^ (corresponding to the C=C bond stretching vibration peak). In addition, these absorption peaks are stronger in ASH-850 and ASH-750 than in SH-850, leading to better adsorption performance. This means that Ni(NO_3_)_2_/KOH as co-catalysts can effectively create more functional groups to enhance the adsorption capacity of CO_2_.

#### 2.1.4. Characterization of the Samples Using X-ray Photoelectron Spectroscopy

**Figure 4:**
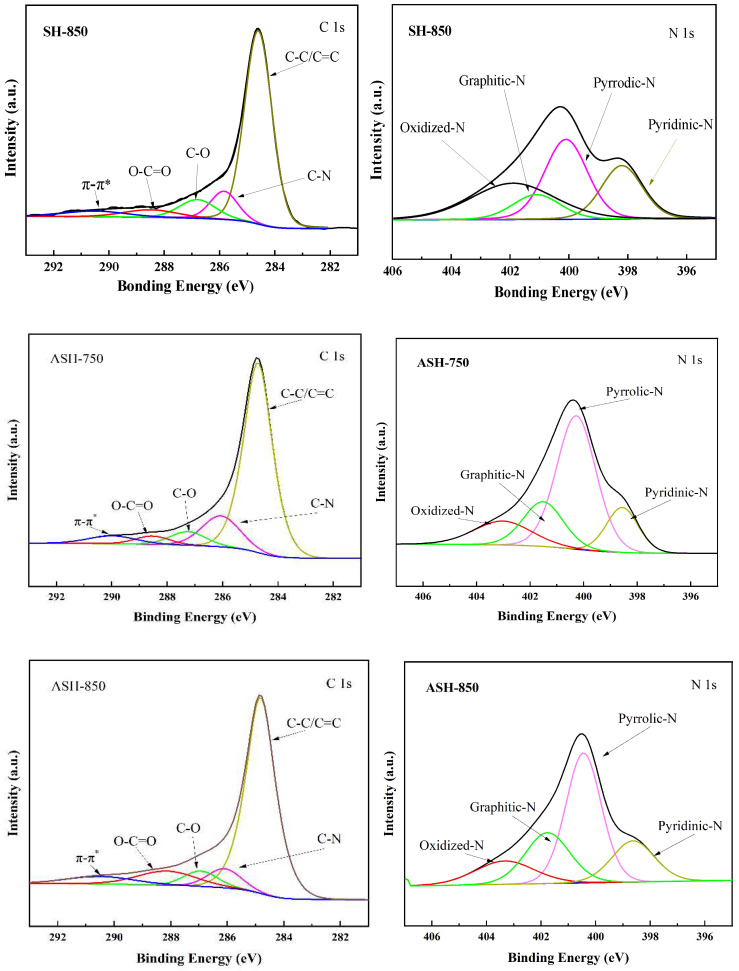
XPS patterns of the SH-850, ASH-750, and ASH-850 samples.

To clarify the effects of Ni(NO_3_)_2_/KOH co-catalysts and activation temperature on the chemical composition, X-ray photoelectron spectroscopy was performed to investigate the surface chemical ingredients and analyze the surface functional groups of the SH-850, ASH750, and ASH-850 samples. Figure 4 shows the full-scan spectra, which indicate that all samples mainly consist of C, N, and O. The significant changes indicate that not only activation temperature can regulate the surface composition but it also has a synergistic effect with the Ni(NO_3_)_2_/KOH dose. As shown in Table 4, the relative percentages of pyridinic-N increased from 14.59% to 17.51%, whereas the relative percentages of pyrrolic-N gradually decreased from 53.28% to 46.08%, when the activation temperature rose. On the other hand, the relative percentages of graphitic-N also increased from 18.20% to 22.62% with a rise in activation temperature, which indicated that higher activation temperatures could promote graphitization. This is also consistent with the findings of our previous study [11]. Compared to the SH-850 and ASH-850 samples, the synergistic effect of Ni(NO_3_)_2_/KOH is mainly reflected in the contents of graphitic-N (increased from 16.54% to 22.62%) and oxidized-N (decreased from 19.32% to 13.79%). The increase in graphitic-N in ASH-850 intensified the spin–orbit coupling structure of the carbon material because of the charge-compensated n-p co-doping, which overcame the main shortcoming arising from single-element adsorption in the carbon material. Figure 4 shows the fitted high-resolution C1s and N1s spectra of these samples. The C1s spectrum was resolved into five peaks, centered at 284.6 eV, 285.7 eV, 286.8 eV, 288.5 eV, and 290.5 eV, which represent C-C/C=C, C-N, C-O, O-C=O bonds, and the π-π* shake-up line, respectively. For the N1s spectrum, four peaks were assigned to pyridinic-N at 398.2 eV, pyrrolic-N at 400.1 eV, graphitic-N at 401.1 eV, and oxidized-N at 401.9 eV. Compared to the synergistic effect of Ni(NO_3_)_2_/KOH, the changes in the surface nitrogen-containing functionalities relied more on the activation temperature (Table 4). As shown in Figure 4, with an increase in activation temperature, the content of pyrrolic-N decreases while the contents of pyridinic-N and oxidized-N increase according to the N1s spectrum, implying that pyrrolic-N was transformed into pyridinic-N and oxidized-N. At the same time, the peak area of C-N is obviously reduced in the C1s spectrum, which is mainly due to the unstable volatilization of nitrogen at higher temperatures.

#### 2.1.5. Characterization of the Samples Using X-ray Diffraction

**Figure 5:**
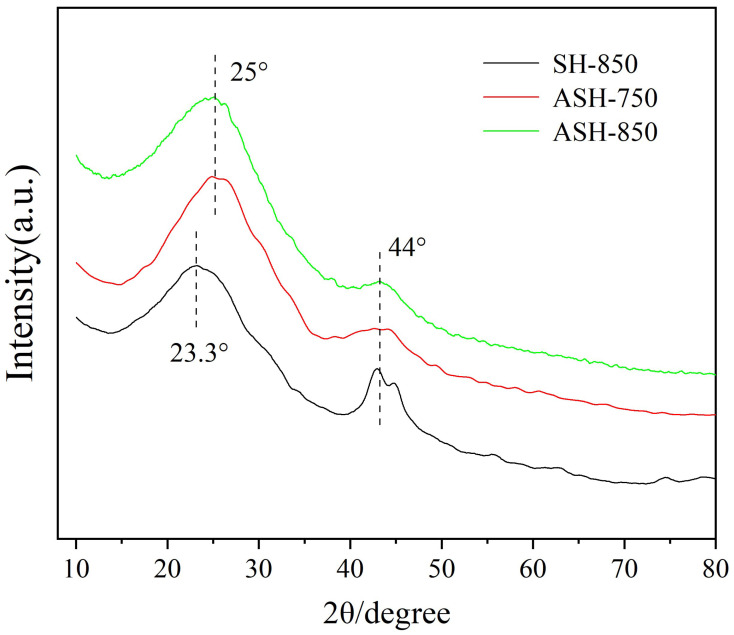
XRD patterns of the SH-850, ASH-750, and ASH-850 samples.

The XRD patterns of the SH-850, ASH-750, and ASH-850 samples are shown in Figure 4. All samples exhibit two peaks at about 2θ ≈ 24° and 44°, corresponding to the diffraction from the (002) planes and (101) planes according to the standard pattern for carbon. The position of this peak is always affected by the activation temperature and catalyst addition, consequently leading to the expansion of the carbon lattice, random distribution of the aromatic carbon sheets, and decomposition of ordered structures on the surface of the carbon lattice [29]. Figure 4 shows that ASH-850 and ASH-750 both yield a stronger peak (002) than SH-850, which implies that ASH-850 and ASH-750 both have better crystallinity with very small crystals. These results clearly indicate that significant changes in the crystal structure and grain size have occurred; furthermore, the peak at about 2θ = 44° of ASH-850 and ASH-750 becomes flat and free of miscellaneous peaks, which implies that the degree of ordered structures in ASH-850 and ASH-750 has increased. In addition, the upside angle of ASH-750 and ASH-850 is both bigger than SH-850 when 2θ < 10° in Figure 5, and this is attributed to the abundant pores in the activated sample, which is in accordance with the SEM characterization and N_2_ adsorption–desorption isotherm results.

#### 2.1.6. Characterization of the Samples Using Raman Spectroscopy

**Figure 6:**
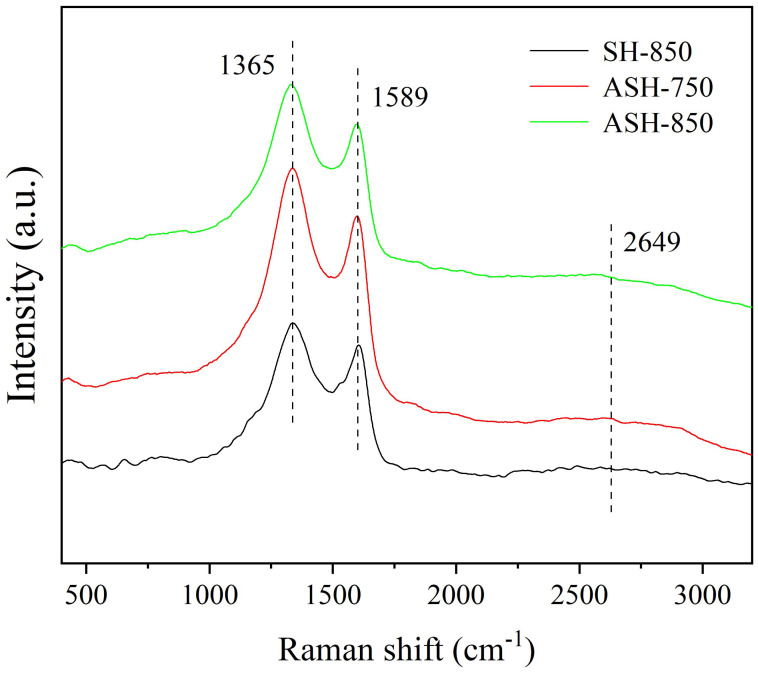
Raman spectrum of the SH-850, ASH-750, and ASH-850 samples.

The results of the Raman spectroscopy are shown in Figure 6. The Raman spectrum shows that both ASH-750 and ASH-850 samples have a typical partly disordered graphitic system with three band features, namely the D band (~1365 cm ^−1^), the G band (~1589 cm ^−1^), and the 2D band (~2649 cm^−1^). The D band located at 1340 cm^−1^ is attributed to the disordered graphite with A_1g_ symmetry. The G band centered at 1580 cm^−1^ corresponds to the first-order scattering of the E_2_g mode of sp^2^ carbon domains. Meanwhile, the intensity ratio of the G band to the D band (ID/IG) of ASH-750, ASH-850, and SH-850 is 1.56, 1.26, and 1.18, respectively, which means that ASH-750 and ASH-850 contain more graphitized structures than disordered carbon. The results indicate that the co-catalyst Ni(NO_3_)_2_/KOH has positive effects at 750 °C and 850 °C, which are much lower than the conventional graphitization temperature (2500–3000 °C) [30,31]. The 2D peak is a prominent feature of graphene in Raman spectra, and its position and shape can be used to distinguish single-layer, bilayer, or few-layer graphene. In this study, both ASH-750 and ASH-850 show a weak 2D band, although it is much broader than single-layer graphene, while SH-850 shows no 2D band. This reveals that porous, activated carbon can be formed in a laminate type of structure using SH as the precursor, followed by activation with Ni(NO_3_)_2_/KOH at lower temperatures.

### 2.2. Characters of CO_2_ Capture

#### 2.2.1. CO_2_ Uptake Capacity

The CO_2_ uptake capacities of SH-850, ASH-750, and ASH-850 were examined at 30 °C, 45 °C, and 60 °C, respectively. ASH-850 showed higher CO_2_ adsorption capacities of 110.35 mg·g^−1^, 94.09 mg·g^−1^, and 72.62 mg·g^−1^, which were higher than ASH-750 at 1 bar, as shown in Table 5. This is due to the fact that the CO_2_ uptake of activated carbon was mainly determined by the high ultra-micropore volume. Though ASH-750 had a larger total porous volume, its further CO_2_ adsorption was limited as a consequence of its lower micropore volume. Previous studies showed that when the pore size of the adsorbent was two times larger than the kinetic diameter of the adsorbate molecule, the highest adsorption energy could be reached [31]. The kinetic diameter of CO_2_ is 0.33 nm; hence, superior CO_2_ adsorption capacity can be achieved by ASH-850 due to its abundant, narrow micropores of 0.67 nm. This was also confirmed by the adsorption experiments. Additionally, the CO_2_ uptake capacities of ASH-750 and ASH-850 were both superior to SH-850. Meanwhile, the CO_2_ uptake capacity of ASH-850 was approximately the same as ASH-4-850, as shown in Tables S1 and S2. These results illustrate that without Ni(NO_3_)_2_, a dosage of KOH at four times higher is needed to achieve similar BET results and CO_2_ uptake capacity; thus, there is a synergistic effect of Ni(NO_3_)_2_/KOH for preparing high-performance activated carbon.

The Ni(NO_3_)_2_/KOH co-catalyst method exhibited CO_2_ adsorption capacity as high as 110.35 mg/g at 30 °C in ASH-850 and 102.51 mg/g at 30 °C in ASH-750. These samples both had a considerably higher CO_2_ adsorption capacity than other carbon materials, as shown in Table 6. The Ni(NO_3_)_2_/KOH co-catalyst was produced from marine waste via a facile method and was used as a CO_2_ adsorbent to increase its added value, aiming to alleviate *Sargassum horneri* golden tides.

#### 2.2.2. Adsorption Kinetics

**Figure 7:**
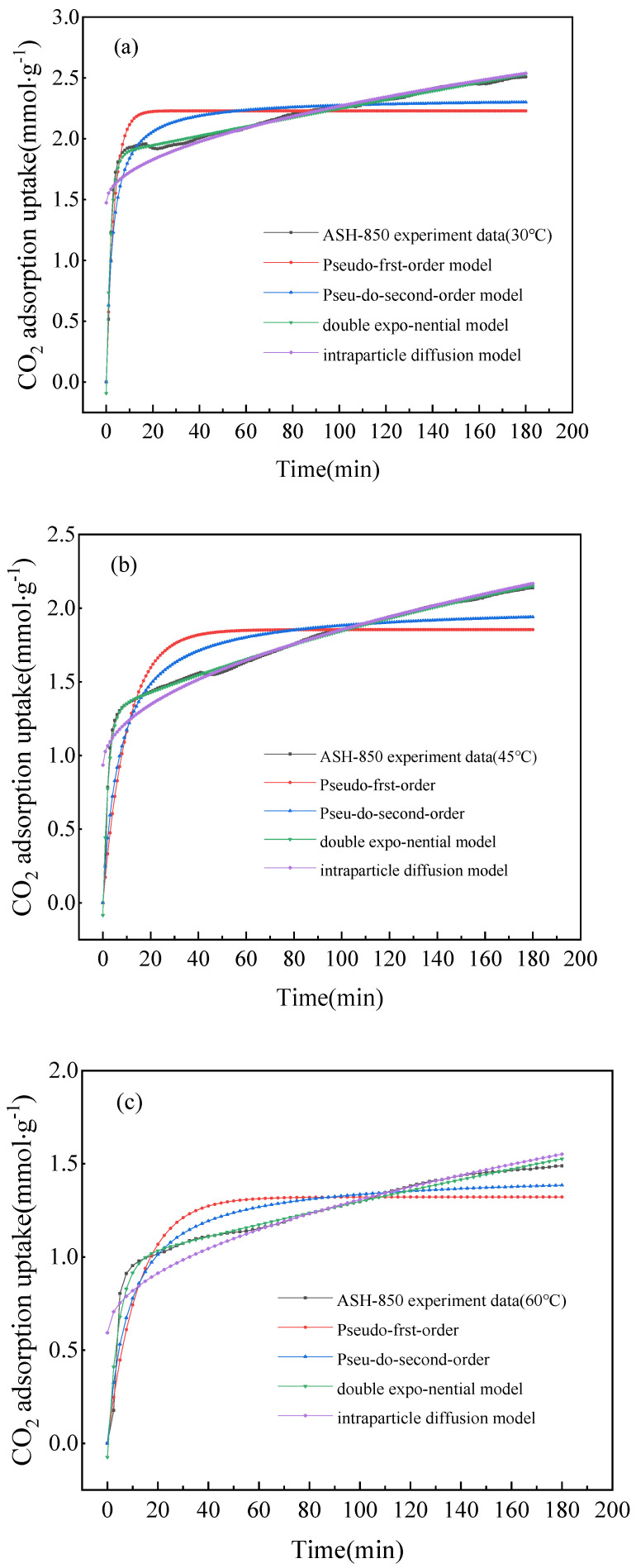
The diagrams of CO_2_ adsorption being fitted using four kinetic models: (**a**) ASH-850 at 30 °C; (**b**) ASH-850 at 45 °C; and (**c**) ASH-850 at 60 °C.

Figure 7 shows that the double exponential model had the best fitness for CO_2_ adsorption capacity of ASH-850 at 30 °C, 45 °C, and 60 °C due to its highest value of R^2^ (>99%), while other methods had values less than 80%; the relevant kinetic parameters are listed in Table 7 and Table 8. Thus, the double exponential kinetic model provided the best description over the entire adsorption process. It suggests that physical adsorption and chemical adsorption occur simultaneously [39]. The almost vertical adsorption curve at the beginning of adsorption illustrates that CO_2_ is rapidly adsorbed within 6 min, which may be because of a good number of active adsorption sites on the surface of the samples. These active sites are probably the functional groups that are shown using FIRT. Gradually, these active sites are occupied, leading to a decreased adsorption rate. As adsorption continues, CO_2_ begins to diffuse inside the particles until the adsorption process reaches an equilibrium.

#### 2.2.3. Adsorbent Regeneration

**Figure 8:**
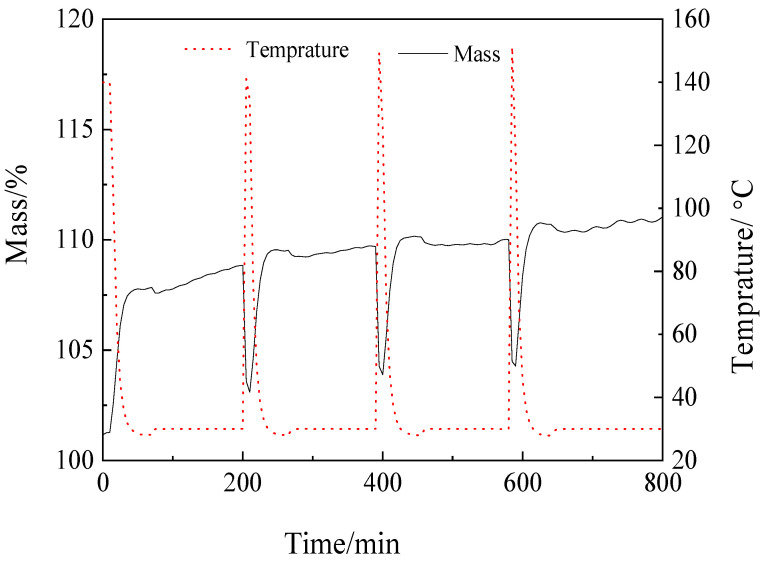
CO_2_ adsorption–desorption cycles obtained for ASH-850 at 30 °C and 1 bar.

Aside from high CO_2_ adsorption capacity, cyclic stability and ease of regeneration are also important criteria for efficient CO_2_ capture in practical applications. A cyclic test was performed by alternatingly repeating the adsorption–desorption cycles at 30 °C and 1 bar, as shown in Figure 8. It can be seen that adsorbed CO_2_ is easily desorbed via purging with N_2_. In fact, more than 93% of CO_2_ could be desorbed within 3 min under desorption conditions. Besides that, no noticeable decrease, or even an increase, in the CO_2_ adsorption capacity was observed after four successive cycles of adsorption and desorption. Thus, ASH-850 could be successfully regenerated at 30 °C and 1 bar and showed high cyclic stability, which is highly desirable for potential industrial applications.

## 3. Materials and Methods

### 3.1. Materials

Nickel (II) nitrate hexahydrate (CAS 13478-00-7 99.5%, wt.%), potassium hydroxide (CAS 1310-58-3, ≥99.0 wt.%), and hydrochloric acid (CAS 7641-01-0, 36.0 wt.%) were purchased from Sinopharm Ltd. (Hangzhou, China). N_2_ (CAS 7727-37-9,99.9%) and CO_2_ (CAS 124-38-999.99%) gases were purchased from Hangzhou Special Gas Co., Ltd. (Hangzhou, China). SH was sampled from a coastal area in Wenzhou, Zhejiang province, China. SH was washed with deionized water several times and dried, which was followed by grinder crushing and sieving to select particle sizes in the range of 400–600 μm. The pretreated SH was carefully oven-dried at 105 °C for 12 h to eliminate moisture before use.

### 3.2. Preparation of Activated Carbon

A fixed amount of Ni(NO_3_)_2_·6H_2_O (0.3625 g) was dissolved in water, which was transferred into a crucible with dried SH (5 g). The mixture was placed for 1 h at room temperature, dried at 105 °C for 12 h, and then put into a muffle furnace to be carbonized at 400 °C under an N_2_ atmosphere for 60 min to decompose nitrate hexahydrate. The as-prepared product (2 g) was cooled down and soaked with potassium hydroxide solution (5 mL, 7.5 mol/L) for 1 h. This mixture was then put into an oven, accompanied by system heating up to a fixed temperature (650, 750, 850, 950, and 1050 °C) separately for 1 h with the protection of 300 mL·min^−1^ N_2_ flow. After activation, the device was cooled to collect activated carbon, during which the solid residue was carefully washed with water, filtered, and dried. The generated carbon was again washed with HCl (100 mL, 1 mol/L) at 150 °C to eliminate K species and other metal irons. The final product was stored for characterization and further utilization. The carbon materials prepared from SH were marked with the corresponding activation temperature, such as “ASH-850”. For comparison, activated carbon without nickel nitrate and potassium hydroxide that was prepared using the same procedure at 850 °C was labeled “SH-850”.

### 3.3. Characterization

Elemental analysis (C, H, and N) of SH was characterized using a common elemental analyzer (Vario Macro cube) from Elementar (Hanau, Hessian, Germany). Metal irons in SH were detected using an inductively coupled plasma-source mass spectrometer (Elan DRC-e) from PerkinElmer (Norwalk, CT, USA). N_2_ adsorption–desorption isotherms were measured at 77 K using an automatic surface area and pore size analyzer (3H-2000PS1), and CO_2_ adsorption experiment was performed at 273 K. Prior to the sorption measurements, the samples were degassed at 200 °C for 3 h. The BET surface area and pore volume were determined using the N_2_ adsorption–desorption Hindawi Template version: May isotherms. The specific surface area was calculated using the Brunauer–Emmett–Teller (BET) method at relative pressure (P/P_0_) values ranging from 0.04 to 0.32; the total pore volume (V_t_) was determined based on the adsorption amount at a relative pressure of 0.99; the micropore surface area (S micro) and micropore volume (V micro) were calculated using the t-plot analysis; and the pore-size distributions (PSDs) were obtained using the density functional theory (DFT) method. The surface morphology of microporous carbon was characterized using a Hitachi S-4700 scanning electron microscope (SEM) at 5.0 kV and 15.0 kV and a Tencnai G2 F30 S-Twin transmission electron microscope (TEM) at 300 kV. The infrared spectra of the samples were acquired using a Nicolet 6700 (FTIR) spectrometer by averaging 24 scans in the 4000–400 cm^−1^ spectral range at 4 cm^−1^ resolution, and a KBr pellet was used as the reference sample. The XRD patterns were collected using a PANalytical X’Pert PRO diffractometer with Cu-Kα radiation (40 kV, 40 mA). The Raman spectra were obtained using a LabRam HR UV 800 Laser Raman Micro spectral probe with an excitation wavelength at 632.81 nm via a diode-pumped solid-state laser. X-ray photoelectron spectroscopy was used to detect the elemental composition and electron valence states on the surface of the samples using a ThermoFischer’s ESCALAB 250Xi X-ray photoelectron spectrometer (Waltham, MA, USA). The excitation source was an Al Kα (1486.6 eV) ray, and the binding energy was calibrated with C1s at 284.6 eV.

### 3.4. CO_2_ Adsorption Experiments

The CO_2_ adsorption performance of the carbon samples was measured using a thermogravimetric analyzer. Initially, about 10 mg of each sample was placed in an alumina crucible loaded in a TGA furnace. Prior to each adsorption experiment, the carbon sample was heated up to 130 °C (10 °C/min) and kept for 30 min to remove moisture under N_2_ flow (40 mL·min^−1^). Then, the carbon sample was cooled to a desired adsorption temperature, i.e., 30 °C, 45 °C, and 60 °C, under which the CO_2_ adsorption studies were performed for 180 min with rate of 50 mL·min^−1^. Moreover, adsorbent regeneration was carried out by heating the sample to 140 °C for 30 min at 10 °C·min^−1^ under N_2_ flow (40 mL·min^−1^). To check the adsorbent stability, the adsorption–desorption procedure was repeated 4 times.

### 3.5. CO_2_ Adsorption Kinetic Analysis

Four typical kinetic models, namely, pseudo-first-order model [40,41] (used to describe the physical adsorption process), pseudo-second-order model [40,42,43] (used to describe the chemical adsorption process), double exponential model [42] (used to describe both the physical and chemical adsorption processes), and intraparticle diffusion model [44] (used to describe the adsorption process in a solid adsorbent with abundant pores), were studied in this research. The regression coefficient (R^2^) was verified according to the fitting degree of each theoretical model and the actual data. The highest regression coefficient indicates the most appropriate theoretical model, which can demonstrate the adsorption kinetic mechanism. The equations for each model are displayed in order as follows:

(6) $$q_{t}=q_{e}\left(1-e^{-k_{1}t}\right)$$

(7) $$q_{t}=\frac{q_{e}^{2}k_{2}t}{1+q_{e}k_{2}t}$$

(8) $$q_{t}=q_{e}-A_{1}\exp\left(-k_{3}t\right)-A_{2}\exp\left(-k_{4}t\right)$$

(9) qt=k5t12+C

where *t* (min) is the time of adsorption; *q*_t_ (mmol·g^−1^) and *q*_e_ (mmol/g) are the amounts of solute adsorbed at time t and at saturation; *k*_1_ (min^−1^) and *k*_2_ (g·mmol^−1^·min^−1^) are the rate constants for pseudo-first-order and pseudo-second-order adsorption, respectively; *k*_3_ (min^−1^) and *k*_4_ (g·(mmol min) ^−1^) are the adsorption rate constants of the first and second adsorption mechanisms in the double exponential kinetic expression, while *k*_5_ (mol/(g·s^0.5^)) is the intraparticle diffusion rate constant; A_1_ (mmol·g^−1^) and A_2_ (mmol·g^−1^) can be viewed as the maximum adsorption capacity in the double exponential model; and *C* is a constant for the intraparticle diffusion model, which is related to the boundary layer’s thickness.

## 4. Conclusions

In summary, we reported a facile method for preparing porous graphitized carbon from SH via Ni(NO_3_)_2_/KOH co-catalysts, which reduces the dosage of the activating agent and lowers the reaction temperature. Compared to ASH-750, ASH-850 presented a higher specific surface area (1486 cm^3^·g^−1^), an extremely higher micropore volume (0.74 cm^3^·g^−1^), and more abundant functional groups, which led to an outstanding capacity of CO_2_ capture. The highest CO_2_ uptake of 2.51 mmol/g was achieved at 30 °C and 1 bar. Moreover, ASH-850 exhibited a rapid CO_2_ adsorption rate, excellent cyclic stability, and easy regeneration. In addition, the kinetic study at different temperatures indicated that both physical adsorption and chemisorption existed during the process of adsorption of the samples. This facile and cost-effective carbon synthesis route is beneficial to large-scale preparation of adsorbents of CO_2_. It also plays an important role in improving the high-value-added utilization of macroalgae, which promotes the development of ecological restoration using macroalgae.

## Figures and Tables

**Table 1:** Elemental analysis of SH and other biomasses.

Biomass	C (wt.%)	H (wt.%)	N (wt.%)	K (mg·kg^−1^)	Reference
SH	29.25	5.96	3.29	104,007.6	This work
*Enteromorpha*	27.85	5.53	2.70	92,740	[25]
*Laminariadigitata*	28.23	3.73	2.31	-	[26]
Beech	49.10	5.70	0.15	-	[26]
Populus wood	46.61	6.32	0.25	4300	[27]

**Table 2:** Elemental analysis of the samples.

Samples	C (wt.%)	H (wt.%)	N (wt.%)
SH	29.25	5.96	3.29
SH-850	81.82	1.37	2.54
ASH-750	76.21	2.36	3.19
ASH-850	84.24	0.92	1.72

**Table 3:** Textural properties of the samples.

Sample	S_BET_/m^2^·g^−1^	V_total_/cm^3^·g^−1^	V_micro_/cm^3^·g^−1^	Average Pore Size/nm
SH	0.43	0.01	0.00	21.77
SH-850	133.53	0.14	0.06	4.30
ASH-650	280.81	0.29	0.14	4.11
ASH-750	1307.30	2.27	0.64	6.97
ASH-850	1486.38	0.93	0.74	2.51
ASH-950	1149.20	0.87	0.47	3.04
ASH-1050	1071.41	1.13	0.39	4.20

**Table 4:** Content of surface nitrogen species of the samples after fitting the N1s peak.

Sample	Pyridinic-N (%)	Pyrrolic-N (%)	Graphitic-N (%)	Oxidized-N (%)
SH-850	17.39	46.75	16.54	19.31
ASH-750	14.59	53.28	18.20	13.93
ASH-850	17.51	46.08	22.62	13.79

**Table 5:** CO_2_ equilibrium adsorption capacities of the samples at different temperatures.

Samples	CO_2_ Adsorption Capacity (q_max_)/mg·g^−1^ (mmol·g^−1^)		
	30 °C	45 °C	60 °C
SH-850	45.32 (1.03)	30.80 (0.70)	28.60 (0.65)
ASH-750	102.51 (2.33)	91.09 (2.07)	68.63 (1.56)
ASH-850	110.35 (2.51)	94.09 (2.13)	72.62 (1.65)

**Table 6:** Comparisons of adsorbent capacity.

Material	T/K	CO_2_ Adsorption Capacity/mg·g^−1^ (mmol·g^−1^)	Ref.
Porous graphene nanosheet	298	101.2 (2.30)	[32]
Mesoporous sucrose-based activated	298	76 (1.7)	[33]
Metal-rich, wood-based activated carbon	298	83 (1.9)	[34]
A material composed of zeolite and activated carbon	298	116 (2.63)	[35]
Sargassum horneri-based porous carbon	303	101.64 (2.31)	[10]
Β-Zeolite	303	77.44 (1.76)	[36]
Waste ion-exchange resin-based activated carbon	303	81.2 (1.8)	[37]
Siliceous zeolites	303	52.8 (1.20)	[38]
ASH-750	303	102.51 (2.50)	This work
ASH-850	303	110.34 (2.13)	This work

**Table 7:** The kinetic parameters of CO_2_ adsorption on activated carbon.

Kinetic Parameters	Kinetic Models			
	Pseudo-First-Order Kinetic Model	Pseudo-Second-Order Kinetic Model	Double Exponential Model	Intraparticle Diffusion Model
*q*_e_ (mmol·g^−1^)	7.0234	7.3565	13.5715	
*k*	k_1_ = 0.2981 (min^−1^)	k_2_ = 0.0504 (mmol∙min^−1^)	k_3_ = 0.5515 (min^−1^) k_4_ = 0.0018 (mmol∙min^−1^)	k_5_ = 0.2500 (mmol∙(g·s^0.5^)^−1^)
A (mmol∙g^−1^)			A_1_ = 6.1462 A_2_ = 7.7120	
C				4.6431
R^2^ (%)	62.8624	79.1138	99.1483	74.6191

**Table 8:** The kinetic parameters of CO_2_ adsorption of ASH-850 at 30 °C, 45 °C, and 60 °C after fitting using the double exponential model.

Double Exponential Model Parameter	CO_2_ Adsorption Temperature		
	30 °C	45 °C	60 °C
*q*_e_ (mmol·g^−1^)	2.53	2.15	1.68
*k*_3_ (min^−1^)	0.5515	0.4699	0.247832
*k*_4_ (mmol∙min^−1^)	0.0018	0.0039	0.002529
A_1_ (mmol∙g^−1^)	6.1462	5.9662	3.387032
A_2_ (mmol∙g^−1^)	7.7120	7.2156	4.997272
R^2^ (%)	99.1483	99.5432	99.0814

## Data Availability

The data used to support the findings of this study are included within the article.

## Supplementary Materials

The following supporting information can be downloaded at: https://www.mdpi.com/article/10.3390/molecules28176242/s1, Table S1. Textural properties of Samples. Table S2. CO_2_ equilibrium adsorption capacities of carbon materials at 30 °C.

## Sample Availability

Samples of the compounds are available from the Huijuan Ying in one year.
