# Supplementary material for: Enhanced Assembling of N-and-K-Riched Macroalgae as Carbon Adsorbent for CO2 Capture with Ni(NO3)2/KOH as Co-Catalysts
Source: Molecules. 2023 Aug 25;28(17):6242. doi: 10.3390/molecules28176242 (PMC10488466; doi:10.3390/molecules28176242)
Supplement: Supplementary file 1 [file molecules-28-06242-s001.zip › molecules-2485535-supplementary.pdf]

Table S1. Textural properties of Samples.

Sample	S_{BET} /m^2·g^{-1}	V_{total} /cm^3·g^{-1}	V_{micro}/ cm^3·g^{-1}	Average pore sizes/nm
SH	0.43	0.01	0.00	21.77
SH-850	133	0.14	0.06	4.30
ASH-850	1486	0.93	0.71	2.51
ASH-4-850	1411	1.16	0.63	3.30

Remark: ASH-4-850 sample was prepared with 4 times dosage of KOH as ASH-850 under the same condition but without Ni $(NO_3)_2$ (the relevant data was published in our paper: Ai, Ning, Sa Lou, Fengyan Lou, Chengda Xu, Qining Wang, and Ganning Zeng. Facile synthesis of macroalgae-derived graphene adsorbents for efficient CO_2 capture. Process Safety and Environmental Protection 2021, 148: 1048-1059.)

Table S2. CO_2 equilibrium adsorption capacities of carbon materials at 30 °C.

Samples	CO_2 adsorption capacity(q_{max}) /(mmol·g^{-1})			
	SH-850	ASH-750	ASH-850	ASH-4-850
**30 °C**	1.03	2.33	2.51	2.78

Remark: Compared with SH-850, ASH-750 aand ASH-850, the CO_2 uptake capacities of ASH-750 and ASH-850 were both superior to SH-850. It illustrated that Ni $(NO_3)_2$ /KOH as a co-catalyst can prepare adsorbent with good performance; Compared with ASH-850and ASH-4-850, it can be seen that without Ni $(NO_3)_2$, four times dosage of KOH was needed to achieve the similar BET and results. Thus there is a synergistic effect of Ni $(NO_3)_2$ /KOH for preparing high-performance activated carbon.
